# Nestin Expression Is Associated with Relapses in Head and Neck Lesions

**DOI:** 10.3390/diagnostics11040583

**Published:** 2021-03-24

**Authors:** Mario Pérez-Sayáns, Cintia M Chamorro-Petronacci, Fátima Baltazar, Fabio Ramoa Pires, Ángel Ínsua, Juan A Suárez-Quintanilla, José M Suárez-Peñaranda

**Affiliations:** 1 Oral Medicine, Oral Surgery and Implantology Unit (MedOralRes), Faculty of Medicine and Dentistry, Universidade de Santiago de Compostela, 15782 Santiago de Compostela, Spain; 2 Research Institute of Santiago de Compostela (IDIS), 15706 Santiago de Compostela, Spain; cintiamica.chamo@yahoo.es; 3 Life and Health Science Research Institute (ICVS), School of Medicine University of Minho, 4710-057 Braga, Portugal; fbaltazar@med.uminho.pt; 4 Post-Graduation Program in Dentistry, Department of Oral Pathology, Estácio de Sá University, State University of Rio de Janeiro, Rio de Janeiro 22631-000, Brazil; ramoafop@yahoo.com; 5 Department of Periodontics and Oral Medicine, University of Michigan, Ann Arbor, MI 48109-1078, USA; angel_insua@yahoo.es; 6 Area of Human Anatomy and Embryology, Faculty of Medicine and Dentistry, University of Santiago de Compostela, 15782 Santiago de Compostela, Spain; juanantonisuarez.suarez@usc.es; 7 Pathological Anatomy Service, University Hospital Complex of Santiago (CHUS), 15706 Santiago de Compostela, Spain; jm.suarez.penaranda@gmail.com

**Keywords:** head and neck neoplasms, nestin, immunohistochemistry, recurrence

## Abstract

Background: The aim was to investigate the clinical significance of nestin immunohistochemical expression in head and neck area lesions and to study its role in patient survival and recurrence. Methods: 39 (44.3%) nasosinus, 37 (42%) major salivary gland (6 submandibular and 31 parotid) and 12 (13.6%) oral cavity lesions of paraffin-embedded samples were retrospectively included. Results: The expression was categorized into grades, negative for 55 (62.5%) cases, grade 1 in 10 cases (11.4%), grade 2 in 12 cases (13.6%), and grade 3 in 11 cases (12.5%); 100% of pleomorphic adenomas were positive for nestin with grade 3 intensity, 100% of polyps and inverted papillomas were negative (*p* < 0.001). The lowest estimate of disease-free-survival (DFS) was for grade 1 expression, with 50 months, confidence interval (CI): 95% 13.3–23.9 months and the highest for grade 3 expression, 167.9 months (CI: 95% 32.1–105 months; Log-Rank = 14.846, *p* = 0.002). ROC (receiver operating characteristic) curves revealed that the positivity for nestin (+/−) in relation to malignancy, presented a sensitivity of 50.98%, a specificity of 81.08%, with an area under the curve of 0.667 (*p* = 0.009). Conclusions: Nestin could be a useful marker to detect the presence of stem cells in head and neck tumors that have a role in tumor initiation and progression.

## 1. Introduction

Nestin is a class VI embryonic intermediary filament protein and has been shown to be a marker of immature/stem cell-like tissue, such as the brain [1], arterial vessels [2], liver [3], major [4] and minor [5] salivary glands, maxillary sinus mucosa [6] and oral mucosa [7,8]. A recent work has related nestin to the phenomenon of epithelial-mesenchymal transition (EMT) and Wnt/beta-catenin signalling [9]. Malvi and Cols. [10] have found that, in mixed primary liver tumors, the hepatocellular-colangiocellular variety were nestin-negative while intermediate-cell variety carcinomas, showed immunoreactivity in most cases (92.3%), which can be useful in tumor differential diagnosis. Intermediary filament (keratins, vimentin and nestin) have been postulated to act as tumoral and metastasis development effectors, following the “hallmarks of cancer” described by Hanahan and Weinberg, keeping cell stemness through CDK5 inhibition [11,12].

During embryogenesis, nestin is expressed in cellular migration and proliferation. By contrast, in adult tissues nestin is restricted mainly to regenerative areas, being abundant in progenitor cells derived from embryonic stem cells, which have the potential to develop into neuroectodermal, endodermal and mesodermal lineages, indicating regenerative potential [13]. It is intensely expressed in cancer cells with high metastatic capacity [14] and, although it is demonstrated that nestin expression inhibition can reduce tumor cell metastatic capacity [15], the metastatic mechanism of this protein in cancer development is still not clear.

Nestin blockade has been shown to inhibit the proliferation of colorectal cancer cell lines [16], migration, invasion and stemness of lung adenocarcinoma lines [14] and a reduction in the 5-year survival rate has been verified in nestin-positive breast cancer patients [17]. Nevertheless, studies in head and neck tumors are scarce. In a systematic review by Curtarelli et al. in 2018 [18], only two studies were included [19,20].

Given the lack of studies on nestin expression in head and neck conditions, the aim of this work is to investigate the clinical significance of nestin immunohistochemical expression in head and neck area lesions (nasosinus, salivary gland and oral cavity) and to study its role in patient survival and recurrence.

## 2. Materials and Methods

Study design: This is a retrospective observational study, designed according to Strengthening the Reporting of Observational Studies in Epidemiology (STROBE) recommendations [21] and approved by the Galician Autonomous Committee of Ethics (reference 2019/271). Samples were retrieved from the registers of the University Hospital Complex of Santiago de Compostela. All procedures were carried out with the understanding and written consent of the subjects in accordance with the Declaration of Helsinki and its subsequent amendments.

Sample selection and histological evaluation: a total of 88 paraffin-embedded samples of benign and malignant conditions with a confirmed histopathological diagnosis were included. Five µm of haematoxylin-eosin stained sections from all cases were histopathologically analyzed and reviewed by two of the authors (JMSP and MPS) who adjusted the diagnosis according to the international recommendations for histopathological analysis. The clinical data of the patients and the characterization of the lesions (AJCC American Joint Committee of Cancer 8th Ed. 2017) were collected in a pseudonymized database.

Immunohistochemical evaluation: immunohistochemistry was performed using a rabbit monoclonal anti-nestin antibody EPR1301(2) (1:500 dilution, Abcam Cambridge, UK) with an automatized equipment (Omnis, Agilent Dako, Santa Clara, CA, USA). In brief, epitope retrieval was performed in 10 mM sodium citrate buffer (pH 9.0) using a water bath for 40 min at 95–99 °C. Endogenous peroxidase was blocked using peroxidase-blocking reagent (Agilent Dako, Santa Clara, CA, USA) for 5 min. Incubation with the primary antibody (1/500) was performed at room temperature for 20 min and staining was revealed with EnVision (20 min) and DAB (diaminobenzidine) 10 min) (Agilent Dako, Santa Clara, CA, USA). Finally, slides were counterstained with HE for 15 min.

Immunohistochemically, only unequivocal cytoplasmic staining was considered positive. Results were evaluated semi-quantitatively attending to the following criteria: negative (Score 0) when no cells stained for nestin; weak (Score 1) when only scattered cells (less than 10% were positive); moderate (Score 2) when positive cells represented 10 to 50%, and strong (Score 3) when over 50% of the cells stained with the antibody. For statistical purposes, nestin expression was also evaluated as negative (score 0) or positive (scores 1, 2 and 3). Stromal or vascular staining was not considered.

Statistical analysis: SPSS v.24.0 (IBM, Statistics, Armonk, NY, USA) was used for statistical analysis. Results were described by mean and standard deviation and frequency with percentages. The data were subjected to the chi-square and Kruskal–Wallis test to study the relationships between the categorical variables. Kaplan–Meier curves were used to study survival and Cox regression with hazard ratio to determine the role of nestin expression. ROC curves were made to estimate the diagnostic efficacy of nestin positivity in malignancy. The significance level was established for *p* ≤ 0.05.

## 3. Results

### 3.1. Clinical and Histopathological Data

A total 88 patients were studied, 40 (45.5 %) women and 48 (54.5 %) men, with an average age of 54.7 ± 18.9 years. The final distribution of the sample was 39 (44.3%) nasosinus, 37 (42%) major salivary gland (6 submandibular and 31 parotid) and 12 (13.6%) oral cavity lesions (Table 1).

Thirty-seven benign (42%) and 51 malignant (58%) lesions were included. The most frequent histological type was carcinoma, with 36 cases (40.9%), followed by inverted papilloma with 14 cases (15.9%) and nasosinus polyps with 13 cases (14.8%). A complete description of different tumor subtypes can be found in Supplementary Information.

### 3.2. Immunohistochemical Expression of Nestin

**Figure 1:**
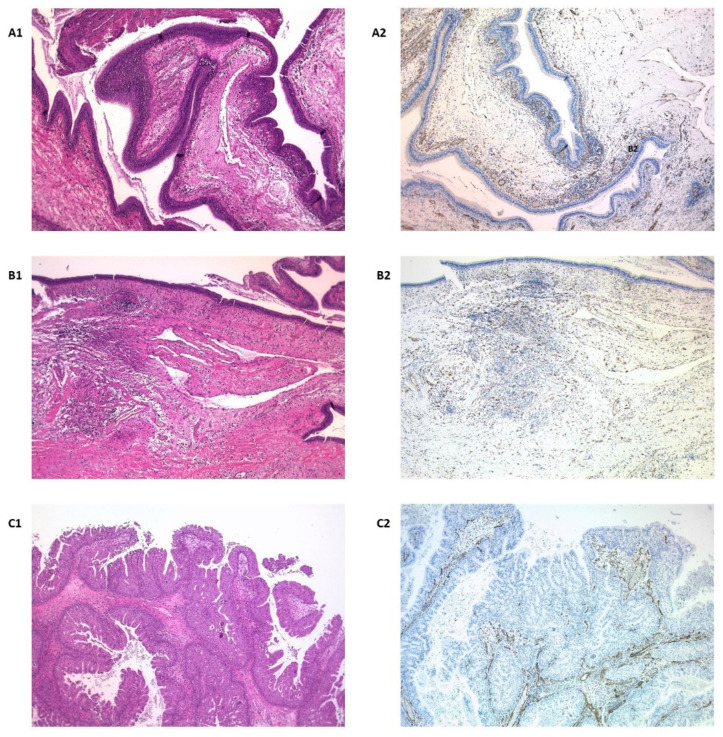
Normal nasosinus mucosa (**A1**) showed nestin expression restricted to vessels and concentrated under the epithelium (**A2**). Inflammatory polyps (**B1**) showed the same staining pattern, consistent with its non-neoplastic nature (**B2**). In inverted nasosinusal papillomas (**C1**), the proliferative epithelium was always completely negative (**C2**).

Nestin staining in normal salivary glands was found to be limited to endothelium and nerve fibers, but parenchymal cells, acinar or ductal, were completely negative except for some myoepithelial cells, which were occasionally stained (Figure 1(A1)).

**Figure 2:**
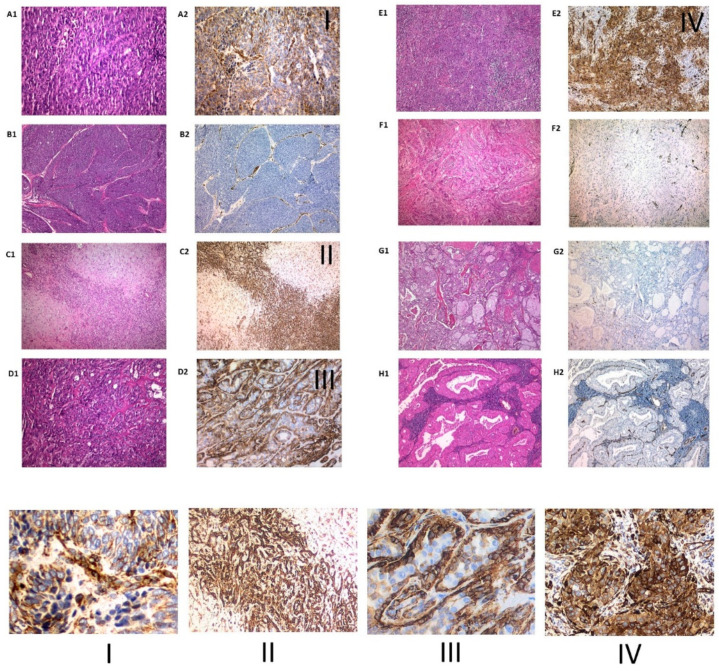
Two examples of poorly differentiated adenocarcinoma, one with extensive immunohistochemical staining (**A1** and **A2**) and another with no nestin expression (**B1** and **B2**). Pleomorphic adenomas (**C1** and **C2**) showed marked and widespread staining for nestin both in the epithelial and mesenchymal components in all cases. In adenoid cystic carcinomas (**D1**) nestin expression was limited to basal cells (**D2**). Squamous cell carcinomas demonstrated a wide range of immunohistochemical staining with some cases strongly positive (**E1** and **E2**) while others were almost completely negative (**F1** and **F2**). Finally, mucoepidermoid carcinomas (**G1** and **G2**) and Whartin (**H1** and **H2**) tumor were always negative. From I to IV, an inset with higher magnification of adenocarcinoma (**I**), pleomorphic adenoma (**II**), adenoid cystic carcinoma (**III**) and squamous cell carcinoma (**IV**).

The expression was categorized into grades, negative for 55 (62.5%) cases, grade 1 in 10 cases (11.4%), grade 2 in 12 cases (13.6%), and grade 3 in 11 cases (12.5%). There were no differences in terms of gender. Regarding origin, 84.6% nasosinus lesions were negative compared to those from the salivary gland and oral origin, which were positive (in different degrees) respectively, in 51.4% and 66.7% (*p* = 0.001). Benign lesions were negative in 81.1% of the cases, against 49% of the malignant ones (*p* < 0.001) (Figure 1(B1,C1)). In relation to the histological type, 100% of pleomorphic adenomas were positive for nestin with grade 3 intensity, 100% of polyps and inverted papillomas were negative (*p* < 0.001) and the rest of the histological varieties were positive in a variable pattern (Figure 2).

### 3.3. Follow-Up: Mortality and Recurrence 

The average follow-up period was 70.5 ± 70.6 months with a range of 1.4 to 280.4 months. 28.4% of the patients died (14.8% due to the tumor), with an overall survival of 43.7 ± 39.9 months with a range of 1.4 to 119.2 months, and the disease specific survival of 38.1 ± 41.6 months, with a minimum of 1.4 months and a maximum of 113.6 months. During the follow-up period, 33% of the lesions recurred, with a mean of 0.6 ± 1.3 recurrences per patient, with a maximum of 9 per patient (adenocarcinoma being the most recurrent variety). The disease-free survival was 39.8 ± 51.6 months, with a minimum of 2.2 months and a maximum of 235.3 months. An average time until the last recurrence of 87.7 ± 74.2 months and an average period of recurrences of 38.8 ± 36.2 months were recorded (Table 2).

In relation to the follow-up in terms of nestin expression (Table 1 and Table 2), no deaths occurred in patients with grade 3 expression, compared to 52% mortality in those who were negative for nestin (*p* < 0.001). In relation to recurrence, the majority of cases are negative (51.7%) compared to cases of intense grade 3 expression in which 6.9% (*p* = 0.038). Grade 1 expression is significantly associated with older patients (76.3 ± 9.4 years, confidence interval (CI): 95% 69.5–83 years, *p* = 0.001). Disease-free survival was significantly lower for lesions with grade 1 expression (15.6 ± 9, CI: 95% 7.2–24 months) than for grade 2 (96 ± 85.5, CI: 95% –10.1–202.2 months) (*p* = 0.001) (Table 2).

### 3.4. Survival Analysis

**Figure 3:**
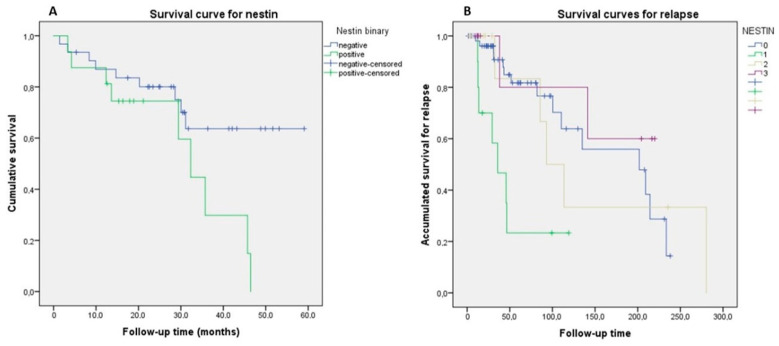
(**A**) Kaplan Meier survival curve designed by excluding pleomorphic adenomas and considering nestin immunohistochemical expression during the first 5 years of follow-up. (**B**) Kaplan Meier survival curve for relapse based on fully graded nestin expression. By means of Cox regression, we verified that the Hazard Ratio for grade 1 expression is 4.577 (confidence interval (CI): 95% 1.802–11.626, *p* = 0001). For a model adjusted by location, sinus lesions are protective for recurrence (hazard ratio (HR) = 0.210, CI: 95% 0.070–0.627, *p* = 0.005) (Table 3).

Kaplan–Meier curves showed that mortality was not a significant factor for the expression of nestin, nevertheless when pleomorphic adenomas were excluded, we considered only the first 60 months of follow-up (5 years), and nestin immunohistochemical expression was not graded and only considered as positive vs. negative, survival was worse for positive nestin cases, 30.41 ± 4.27 months (confidence interval (CI) 95% 22.04–38.77 months) vs. negative cases with 44.48 ± 4.04 months (IC 95% 36.56–52.40 months) (Log-Rank = 3.867, *p* = 0.049) (Figure 3A). For relapses, the lowest estimate of DFS was for grade 1 expression, with 50 months, CI: 95% 13.3–23.9 months and the highest for grade 3 expression, 167.9 months, CI: 95% 32.1–105 months (Log-Rank = 14.846, *p* = 0.002) (Figure 3B).

### 3.5. Diagnostic Yield

**Figure 4:**
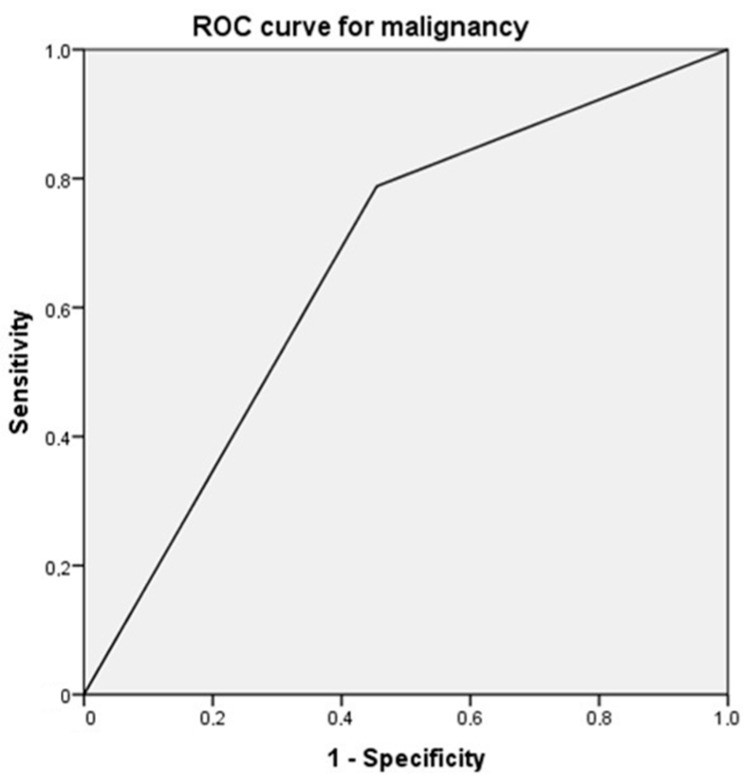
ROC (receiver operating characteristic) curve based on the diagnostic capacity of nestin positivity in relation to malignancy. The area under the curve was 0.667 (*p* = 0.009).

The study using ROC (receiver operating characteristic) curves revealed that the positivity for nestin (+/−) in relation to malignancy (yes/no), presented a sensitivity of 50.98%, a specificity of 81.08%, with an area under the curve of 0.667 (*p* = 0.009) (Figure 4).

## 4. Discussion

We have found a positivity for nestin in head and neck region lesions of 37.5% globally, remaining negative in all inflammatory lesions (polyps) and inverted papillomas) and in 51% of malignant tumour lesions. The malignant lesions in the oral cavity reached a level of positivity of 66.7% significantly, although in relation to intensity, 100% of the pleomorphic adenomas of the salivary gland were grade 3. Mortality and recurrence were higher for negative cases and similarly, the DFS was significantly lower in the cases of grade 1 nestin in relation to the most intense grades. The hazard ratio for recurrence was 4.577 for cases with mild nestin expression (grade 1) and 8918 when we adjusted the model by location.

The intermediate filament protein nestin, initially considered a marker for neural stem cells, is extensively expressed in progenitor cells derived from embryonic stem cells, which have the ability to become multi-linear [13]. Since it is not expressed in mature elements and that terminal cell differentiation is associated with loss of immunoreactivity to this protein, immunohistochemical assessment of nestin expression might be useful to differentiate between mature and immature cells/populations. Some reports have claimed its value to differentiate between benign and malignant lesions [22,23]. Little has been studied about the expression in head and neck entities [18,24]. In a recent meta-analysis conducted by Han et al. [24], on the prognostic value of ALDH1 and nestin in advanced cancers (breast, colon, colorectal, cervical, ovarian, skin and hepatocellular), no head and neck study was included.

It has been reported that expression of nestin in the soft tissues of patients with uni- or bilateral cleft lip may indicate a potential increase of tissue regeneration [25]. Also, nestin staining may even indicate that human oral mucosa can be an effective source for hard tissue regeneration [7] and tooth tissue engineering applications [8]. In relation to the maxillary sinus, the regenerative potential of the Scheneiderian membrane has been postulated, associated with the presence of progenitor cells that express nestin [6,26], but the authors have not found any studies that analyse expression in lesions located in the nasosinus region. Luo et al. [19], studied the expression of embryonic stem cell markers (SOX2, OCT4 and NANOg) in nasopharyngeal lesions, with the aim of analysing EMT. They found that nestin expression was completely absent in non-cancerous epithelium and tumor cells, while it was strongly stained in the cytoplasm of endothelial cells in cancerous tissues, as 32 out of 48 tumors (66.7%) showed cytoplasmic positivity for nestin in the endothelium, frequently located in the invasive front. We have not been able to reproduce these findings and, in our cases, endothelial expression was wide and not limited to the tumor front. Tumor cells exhibiting similar characteristics to cancer stem cells (CSC) significantly correlated with nestin staining on the invasive front, so Luo et al. [19] postulated that vascular endothelial cells expressing nestin may represent the niche of SCC in nasopharyngeal carcinomas. They found no association of nestin with survival. In the present study we have found the same results (Figure 1), being totally negative for inflammatory lesions and with variable positivity in tumor lesions, mainly at endothelial level.

Mascolo et al. [20] studied the expression of molecules associated with resistance to DNA damage (poly(ADP-ribose) polymerase 1 (PARP-1) and chromatin assembly factor-1 (CAF-1)/p60) and of stem cell markers (nestin) in oral squamous cell carcinoma (OSCC) samples. They found that a PARP-1-high/CAF-1-p60-high/nestin-high phenotype characterized OSCC with the worst prognosis, recurrence, metastasis, and death (all HPV-negative). Nestin was particularly expressed in OSCC metastases. The sensitivity of nestin (high expression) to predict at least one adverse event was 100% with a specificity of 56%. In our cohort, we included 11 OSCC and one mucoepidermoid carcinoma of the oral cavity. In the OSCC, 27.3% of the samples were negative for nestin, and expression was intense (grades 2 and 3) in 54.6% of the cases, but we could not establish a relationship with mortality, although to the limited number of cases. In relation to the oral cavity, Kuk et al. [27] found that, in a series of 39 oral melanomas (6 in situ and 36 invasive), found the same as Mascolo et al. [20], this is a relationship of nestin expression (intensity and proportion) and disease progression, as well as a worse prognosis (Hazard ratio 3.59 for intensity and 4.05 for proportion). Ravindran and Devaraj [28] studied the expression pattern and prognostic significance of two neural stem cell markers, nestin and musashi-1, in oral cancer. A gradually increased expression of nestin was found along the transformation stages of oral cancer. Association with higher stage and poor differentiated status of oral carcinomas was identified with nestin or musashi-1 positive lesions. In the same way, a highly significant correlation with poorer survival was detected when both markers were present in the samples.

Regarding salivary gland conditions, Yanai et al. [29] described the expression of nestin in normal glandular tissue and tumors. In normal salivary gland tissue, nestin was detected in the endothelium and nerve fibres [30]. Although most of the luminal and abluminal cells were negative, a few smooth muscle actin-positive myoepithelial cells α from the acini or intercalated ducts expressed nestin. In all pleomorphic adenomas (n = 11), the myoepithelial cells in both the ductal structure and the mesenchymal area showed diffuse staining for nestin, similarly to the grade 3 nestin expression in all pleomorphic adenomas found in the present study. They found no negative adenocarcinomas; however, in our series, 4 (50%) were negative. All mucoepidermoid carcinomas and Whartin’s tumors were negative in both studies, which may serve as a differential diagnostic guide. As previously pointed out, pleomorhic adenomas are the tumor type with the most consistent immunohistochemical expression of nestin. Immunohistochemical expression of some stem cell markers, such as the stem cell marker B cell-specific Moloney murine leukemia virus integration site 1 (Bmi-1) has been reported as an indication of early malignant transformation in pleomorphic adenoma [31]. Nevertheless, none of our cases have behaved aggressively, indicating that nestin is not a marker of malignant transformation in this neoplasm. It is well known that pleomorphic adenomas are characterized by variable epithelial and stromal components in a diversity of patterns. There is undoubtable histopathologically, ultrastructural, immunohistochemical, and molecular evidence to suggest that the mesenchymal elements and the epithelial cells share the same origin [32]. In some areas this neoplasm demonstrates a transitional phenotype with cells showing both epithelial and mesenchymal features, which provides evidence for epithelial-mesenchymal transition as the basic principle of the tissue heterogeneity in pleomorphic adenomas [33]. Consonant with these findings, immunohistochemical overexpression of nestin in pleomorphic adenomas could be interpreted as a sign of this phenomenon instead of being related with the clinical outcome of the neoplasm. Similar results were obtained by Yanai et al. [29] but they did not associate it with the epithelial mesenchymal transition phenomenon. Our results were also similar in adenoid cystic carcinoma, in which expression was strong and constant in peripheral myoepithelial cells, and Warthin tumor and mucoepidermoid carcinoma, both of them completely negative. The role of nestin in the epithelial-mesenchymal transition has already been reported in different contexts [34] and this could represent another example of this function.

In our series we have not been able to demonstrate immunohistochemical expression in any benign neoplasm, except for pleomorphic adenomas, in which it seems to be related with the epithelial-mesenchymal transition and not with the benign or malignant character of the lesion. Instead, malignant tumors have shown different grades of nestin immune expression and, independent of its degree, it could be helpful to assess the malignant character of a lesion in small biopsies. Although overall nestin expression does not seem to be correlated with survival (since in this study all the patients with malignant tumors had died during the large follow-up period), tumor relapsing is associated with nestin staining. Moreover, when pleomorphic adenomas were excluded and nestin immunohistochemical expression was not graded and only considered as positive vs. negative, survival was worse for cases positive for nestin (*p* = 0.049). In head and neck tumors, the vast majority of the stem cells are found within a 100 μm radius of a blood vessel, suggesting the existence of a perivascular niche [35]. It could be argued that in advanced aggressive tumors neoplastic growth overpass their vascular growth capacity, thus limiting the presence or immunohistochemical ability to detect stem cells in some cases. Moreover, tumoral stem cells have proved to be important for tumor development and progression, as well as invasion, chemotherapy resistance, relapses and metastatic dissemination [36] but it is not known if they are always present in the same amount in every step of tumor development or if immunohistochemistry would be effective to detect them after chemotherapy and radiotherapy. Based on the results of recurrence and death, it is our opinion that nestin could be used generally to stratify head and neck cancers. No association has been observed between nestin expression and gender, but it was more commonly expressed in tumors from elderly patients. These results are difficult to interpret because of the different tumor types included in the study and the low number of some of them. More studies including a significant number of cases from each tumoral type would be needed to clarify this issue. Nevertheless, our results confirm that nestin could be a useful marker to detect the presence of stem cells in head and neck tumours, and in fact they have been earlier demonstrated in some types of neoplasm in the area, which may have relevant implications in the management of these neoplasms due to their role in tumor initiation and progression.

## Figures and Tables

**Table 1:** Distribution of categorical variables by Nestin expression analyzed through Kruskal–Wallis test.

Variable		Nestin Expression				Total	*p*-Value
		0	1	2	3		
**Gender**	Female	24 (60)	4 (10)	5 (12.5)	7 (17.5)	40 (45.5)	0.635
	Male	31 (64.6)	6 (12.5)	7 (14.6)	4 (8.3)	48 (54.5)	
**Origin**	Nasosinus	33 (84.6)	2 (5.1)	4 (10.3)	0	39 (44.3)	0.001
	Salivary glands	18 (48.6)	6 (16.2)	3 (8.1)	10 (27)	37 (42)	
	Oral	4 (33.3)	2 (16.7)	5 (41.7)	1 (8.3)	12 (13.6)	
**Localization**	Nasosinus	33 (84.6)	2 (5.1)	4 (10.3)	0	39 (44.3)	0.001
	Parotid gland	16 (51.6)	6 (19.4)	1 (3.2)	3 (25.8)	31 (35.2)	
	Submandibular gland	2 (33.3)	0	2 (33.3)	2 (33.3)	6 (6.8)	
	Alveolar mucosa	3 (60)	0	2 (40)	0	5 (5.7)	
	Buccal mucosa	1 (50)	1 (50)	0	0	2 (2.3)	
	Floor of the mouth	0	0	1 (50)	1 (50)	2 (2.3)	
	Lip	0	0	1	0	1 (1.1)	
	Tongue	0	0	1	0	1 (1.1)	
	Retromolar trigone	0	1	0	0	1 (1.1)	
**Histological type**	Adenocarcinoma	4 (50)	2 (25)	1 (12.5)	1 (12.5)	8 (9.1)	<0.001
	Carcinoma	16 (44.4)	6 (16.7)	11 (30.6)	3 (8.3)	36 (40.9)	
	Lymphoma	1 (33.3)	2 (66.7)	0	0	3 (3.4)	
	Inverted papilloma	14 (100)	0	0	0	14 (15.9)	
	Polyp	13 (100)	0	0	0	13 (14.8)	
	Pleomorphic adenoma	0	0	0	7 (100)	7 (8)	
	Warthin tumor	4 (100)	0	0	0	4 (4.5)	
	Mucoepidermoid carcinoma	3 (100)	0	0	0	3 (3.4)	
**Malignancy**	No	30 (81.1)	0	0	7 (18.9)	37 (42)	<0.001
	Yes	25 (49)	10 (19.6)	12 (23.5)	4 (7.8)	51 (58)	
**Death**	No	42 (66.7)	2 (3.2)	8 (12.7)	11 (17.5)	63 (71.6)	<0.001
	Yes	13 (52)	8 (32)	4 (16)	0	25 (28.4)	
**Death by lesion**	No exitus	42 (66.7)	2 (3.2)	8 (12.7)	11 (17.5)	63 (71.6)	<0.001
	Yes by the lesion	8 (61.5)	2 (15.4)	3 (23.1)	0	13 (14.8)	
	Yes other reasons	5 (41.7)	6 (50)	1 (8.3)	0	12 (13.6)	
**Relapse**	No	40 (67.8)	3 (5.1)	7 (11.9)	9 (15.3)	59 (67)	0.038
	Yes	15 (51.7)	7 (24.1)	5 (17.2)	2 (6.9)	29 (33)	
**Total**		55 (62.5)	10 (11.4)	12 (13.6)	11 (12.5)	88 (100)	

**Table 2:** Distribution of quantitative variables. CI: confidence interval.

	Nestin 0 Average ± SD (CI95%)	Nestin 1 Average ± SD (CI95%)	Nestin 2 Average ± SD (CI95%)	Nestin 3 Average ± SD (CI95%)	*p*-Value
**Age (years)**	51.7 ± 1.5	76.3 ± 9.4	49.5 ± 17.4	55.2 ± 22.3	0.001 *
	(47–56.5)	(69.5–83)	(38.4–60.6)	(40.2–70.2)	
**Overall survival**	40.7 ± 39.9	51 ± 38.4	38.3 ± 51.9	–	0.827
	(16.6–64.9)	(18.9–83.1)	(18.8–83.1)		
**Maximum follow-up time** **Disease Free Survival**	71.73 ± 65.53 54.02–89.45 31.1 ± 39.9 (9–53.2)	43.24 ± 37.41 16.48–70 15.6 ± 9 (7.2–24)	77.80 ± 92.1 19.28–126.32 96 ± 85.5 (–10.1–202.2)	81.47 ± 92.98 19.01–143.94 48.7 ± 37 (–283.8–381.3)	0.600 0.037 **
**Disease specific survival**	37.6 ± 43.3	21.9 ± 13.5	50 ± 56.8	–	0.791
	(1.4–73.8)	(–100–143.9)	(–91.1–191.1)		
**Number of relapses**	0.5 ± 1.3	1.3 ± 1.5	0.75 ± 1	0.27 ± 0.6	0.289
	(0.17–0.92)	(0.18–2.4)	(0.8–1.4)	(–0.16–0.7)	
**Time until last relapse**	84.5–59.3	30.8 ± 6.1	117.9 ± 126	129.4	0.637
	(22.2–146.9)	(–24.5–86.1)	(–195–431)		
**Period of relapses**	53.4 ± 45	13.8 ± 1.37	20.9 ± 16.9	54.52	0.470
	(6.1–100.6)	(1.5–26.1)	(–21–62.9)		

* Global mean difference by analysis of variance (ANOVA) test. Multiple correlations by post-hoc Bonferroni test for groups of nestin expression. * For age: Nestin-0 vs. Nestin-1, *p* = 0.001; Nestin-1 vs. Nestin-2, *p* = 0.003; Nestin-1 vs. Nestin-3, *p* = 0.043. ** For disease-free survival: Nestin-1 vs. Nestin-2, *p* = 0.039.

**Table 3:** A univariate logistic regression analysis was performed to determine the univariate hazard ratio (HR) for Relapse. The statistical analysis of the adjusted HR was performed using gradual Cox Regression Analysis adjusted for Nestin expression and location.

Cox Regression Covariate	Relapse			
	Univariate HR (95% CI)	*p* Value	Adjusted HR (95 % CI)	*p* Value
**Nestin**				
Negative Vs 1	4.577 (1.802–11.626)	0.001	8.918 (2.71–305.62)	<0.001
Negative Vs 2 Negative Vs 3	1.023 (0.332–3.158)	0.968	1.347 (0.354–5.118)	0.662
	0.546 (0.122–2.433)	0.427	0.899 (0.151–5.356)	0.907
**Location**				
Nasal/Sinus Vs trigone	0.034 (0.03–0.389)	0.006	0.210 (0.070–0.627)	0.005
Parotid Vs trigone	0.013 (0.001–0.160)	0.001	0.246 (0.020–2.966)	0.270
Submaxilla Vs trigone	0.010 (0–0.236)	0.004	2.497 (0.413–15.087)	0.319
Alveolar ridge Vs trigone	0.086 (0.006—1.270)	0.074	0	0.992
Buccal mucosa Vs trigone	0	0.991	0	0.994
Floor of mouth Vs trigone	0	0.991	0	0.963
Lip Vs trigone	0	0.956	0	0.995
Tongue Vs trigone	0	0.994	4.405 (0.344–56.384)	0.254

## Supplementary Materials

The following are available online at https://www.mdpi.com/2075-4418/11/4/583/s1, Tumor subtypes.
