# Supplementary material for: Nestin Expression Is Associated with Relapses in Head and Neck Lesions
Source: Diagnostics (Basel). 2021 Mar 24;11(4):583. doi: 10.3390/diagnostics11040583 (PMC8063927; doi:10.3390/diagnostics11040583)
Supplement: Supplementary file 1 [file diagnostics-11-00583-s001.pdf]

diagnostics

Supplementary File

Nestin Expression Is Associated with Relapses in Head and Neck Lesions

Mario Pérez-Sayáns [1,2]*, Cintia M Chamorro-Petronacci [2], Fátima Baltazar [3], Fabio Ramoa Pires [4], Ángel Ínsua [5], Juan A Suárez-Quintanilla [6] and José M Suárez-Peñaranda [7]

[1] Oral Medicine, Oral Surgery and Implantology Unit (MedOralRes), Faculty of Medicine and Dentistry, Universidade de Santiago de Compostela, 15782 Santiago de Compostela, Spain
[2] Research Institute of Santiago de Compostela (IDIS), 15706 Santiago de Compostela, Spain; cintiamica.chamo@yahoo.es
[3] Life and Health Science Research Institute (ICVS), School of Medicine University of Minho, 4710-057 Braga, Portugal; fbaltazar@med.uminho.pt
[4] Post-graduation Program in Dentistry, Department of Oral Pathology, Estácio de Sá University, State University of Rio de Janeiro, Rio de Janeiro 22631-000, Brazil; ramoafop@yahoo.com
[5] Department of Periodontics and Oral Medicine, University of Michigan, Ann Arbor, MI 48109-1078, USA; angel_insua@yahoo.es
[6] Area of Human Anatomy and Embryology, Faculty of Medicine and Dentistry. University of Santiago de Compostela, 15782 Santiago de Compostela, Spain; juanantonisuarez.suarez@usc.es
[7] Pathological Anatomy Service, University Hospital Complex of Santiago (CHUS), Santiago de Compostela, 15706 Santiago de Compostela, Spain; jm.suarez.penaranda@gmail.com.
* Correspondence: perezsayans@gmail.com; Tel.:+34 626233504

Citation: Pérez-Sayáns, M.; Chamorro-Petronacci, C.; Baltazar, F.; Pires, F.R.; Ínsua, Á.; Suárez-Quintanilla, J.A.; Suárez-Peñaranda, J.M. Nestin Expression Is Associated with Relapses in Head and Neck Lesions. *Diagnostics* **2021**, *11*, 583. https://doi.org/10.3390/diagnostics11040583

Academic Editor: Maciej Misiolek

Received: 10 February 2021
Accepted: 21 March 2021
Published: 24 March 2021

Publisher's Note: MDPI stays neutral with regard to jurisdictional claims in published maps and institutional affiliations.

Copyright: © 2021 by the authors. Licensee MDPI, Basel, Switzerland. This article is an open access article distributed under the terms and conditions of the Creative Commons Attribution (CC BY) license (http://creativecommons.org/licenses/by/4.0/).

Tumor subtypes

Mucoepidermoid carcinoma: 8 (9.08%)
Adenoid cystic carcinoma: 6 (6.81%)
Warthin tumor: 4 (4.54%)
Squamous cell carcinoma: 15 (17.04%)
Polyp: 13 (14.77%)
Inverted papilloma: 13 (14.77%)
Inverted papilloma with atypia: 1 (1.13%)
Pleomorphic adenoma: 7 (7.95%)
Parotid squamous cell carcinoma: 1 (1.13%)
Mucosecretory adenoma: 1 (1.13%)
Moderately differentiated adenocarcinoma: 3 (3.4%)
Well-differentiated adenocarcinoma: 1 (1.13%)
Well-differentiated mucinous adenocarcinoma, enteric type: 1 (1.13%)
Moderately differentiated mucinous adenocarcinoma: 2 (2.27%)
Undifferentiated carcinoma trabecular pattern: 1 (1.13%)
Undifferentiated sinonasal carcinoma: 1 (1.13%)
Poorly differentiated adeno squamous carcinoma: 2 (2.27%)
Epithelial-myoepithelial carcinoma: 1 (1.13%)
Large B-cell lymphoma: 1 (1.13%)
Hodgkin lymphoma: 2 (2.27%)
Salivary duct carcinoma: 2 (2.27%)
Small cell carcinoma: 1 (1.13%)
Acinic cell carcinoma: 1 (1.13%)
